# Atherogenic index of plasma and the severity of coronary artery stenosis in patients with type 2 diabetes mellitus: a retrospective cross-sectional study

**DOI:** 10.3389/fendo.2026.1824215

**Published:** 2026-05-29

**Authors:** Juan Liao, Wenzhao Zhang, Liqing Peng, Jing Huang, Gen Chen, Jianqun Yu

**Affiliations:** 1 Department of Medical Imaging, People’s Hospital of Shifang City, Shifang, China; 2 Department of Radiology, West China Hospital, Sichuan University, Chengdu, China

**Keywords:** atherogenic index of plasma, biomarker, cardiovascular risk, coronary artery stenosis, type 2 diabetes mellitus

## Abstract

**Objective:**

This study aimed to examine the relationship between the Atherogenic Index of Plasma (AIP) and the severity of coronary artery stenosis in patients with type 2 diabetes mellitus (T2DM).

**Methods:**

In this retrospective cross-sectional study, 303 T2DM patients who had not been treated with statins and underwent coronary computed tomography angiography (CCTA) were included. AIP was calculated as log_10_(TG/HDL-C). The severity of coronary stenosis was assessed using standard quantitative measures. Participants were divided into four groups based on AIP quartiles (Q1 to Q4). Statistical analyses involved correlation tests, multivariable logistic regression, and receiver operating characteristic (ROC) curve analysis.

**Results:**

A clear dose-response trend was found between increasing AIP quartiles and greater severity of coronary stenosis (P for trend <0.01). The median AIP was significantly higher in patients with severe stenosis (≥70%) compared to those with non-obstructive disease (0.14 vs. 0.05, P<0.001). After adjusting for multiple variables, AIP remained independently associated with obstructive stenosis (≥50%), with an odds ratio of 3.93 (95% confidence interval: 1.70–9.62). The area under the ROC curve (AUC) for AIP was 0.598 (95% CI: 0.540–0.653), which was significantly better than that for LDL cholesterol (0.481, P = 0.032) and HDL cholesterol (0.384, P<0.001).

**Conclusion:**

Higher AIP levels are independently associated with more severe coronary stenosis in patients with T2DM and demonstrate superior discriminative ability compared to traditional lipid markers in this cross-sectional cohort. Therefore, AIP may be considered an adjunctive biomarker for cardiovascular risk stratification in this high-risk population, pending prospective validation.

## 1 Introduction

Cardiovascular disease (CVD) is the leading cause of morbidity and mortality in patients with type 2 diabetes mellitus (T2DM). Those with T2DM face a two- to four-fold higher risk of cardiovascular events compared to people without diabetes, and cardiovascular complications are responsible for most deaths in this group (1). Although current treatments that lower low-density lipoprotein cholesterol (LDL-C) have greatly improved cardiovascular outcomes in T2DM patients (2), clinical observations reveal that even when LDL-C levels are within target ranges, 25% to 40% of patients still experience unexplained residual cardiovascular risk (3). This indicates that factors beyond LDL-C, such as elevated triglycerides and decreased high-density lipoprotein cholesterol (HDL-C), may significantly contribute to the development of atherosclerosis in T2DM patients.

The atherogenic index of plasma (AIP), calculated as the base-10 logarithm of the ratio of triglycerides to HDL-C [log_10_(TG/HDL-C)], has recently garnered widespread attention due to its unique pathophysiological significance (4). Unlike traditional lipid parameters, AIP more accurately reflects the dynamic balance between pro-atherogenic lipoprotein remnants (such as VLDL and IDL) and anti-atherogenic HDL (5). Basic research showed that a higher AIP is not only associated with an increase in small, dense LDL particles (6) but also reflects vascular endothelial dysfunction and chronic low-grade inflammation (7, 8), both of which are key processes in the progression of atherosclerosis.

In T2DM patients, insulin resistance leads to lipid metabolism disturbances characterized by increased triglycerides and decreased HDL-C. This specific pattern of dyslipidemia makes AIP a potentially valuable marker for evaluating cardiovascular risk (9). Multiple cross-sectional studies have identified an association between AIP and coronary artery disease (CAD). For instance, a study involving 3,278 T2DM patients found that AIP independently correlates with CAD and offers better predictive power than traditional lipid markers (10). Another study focusing on patients after percutaneous coronary intervention revealed that higher AIP levels were significantly linked to a greater risk of in-stent restenosis (11).

Recent research has further enhanced understanding of AIP. Zhang and colleagues (12) reported that AIP was positively associated with T2DM risk in a Chinese cohort (HR = 4.40). Wen et al. (13) demonstrated that both baseline AIP and 5-year changes in AIP were associated with T2DM risk, with a 20% risk reduction in those whose AIP decreased over time. Min et al. (14) found that consistently high AIP was associated with increased CVD incidence in patients with abnormal glucose metabolism (OR = 1.56). Bai et al. (15) further showed a non-linear association between AIP and WHO-defined CVD high-risk status (AUC = 0.557, improving to 0.650 after adjustment). Importantly, AIP has advantages over conventional lipid measures in Asian populations, who often exhibit elevated triglycerides and low HDL-C despite normal LDL-C levels—a pattern captured by AIP but overlooked by LDL-C alone (16). Therefore, AIP serves as a practical and cost-effective biomarker derived from routine lipid tests for assessing cardiovascular risk in Asian populations.

However, most prior research has primarily examined the relationship between AIP and the presence of coronary artery disease (CAD), without sufficiently capturing the progressive nature of disease severity. Moreover, within the specific cohort of patients with T2DM, the optimal AIP threshold and its clinical implications have yet to be clearly established. This study aims to assess and quantify the association between AIP and the extent of coronary artery stenosis in T2DM patients using a retrospective cross-sectional approach. Additionally, it aims to determine the optimal AIP cutoff value for identifying clinically significant coronary stenosis, thereby providing a theoretical foundation for cardiovascular risk stratification and individualized therapeutic interventions in this patient population.

## 2 Materials and methods

### 2.1 Study participants

**Figure 1:**
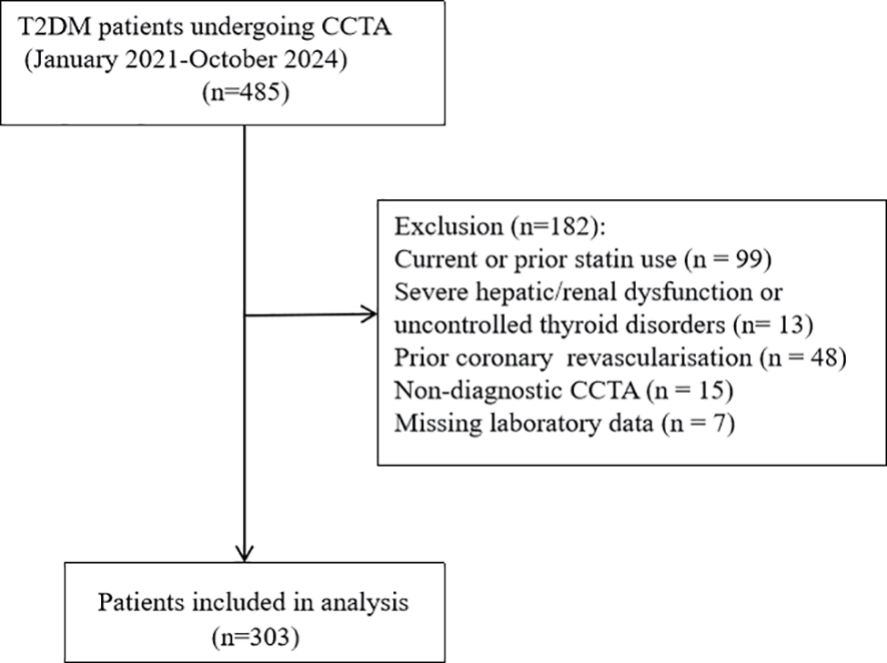
Study flowchart. A total of 485 T2DM patients who underwent CCTA were initially screened. After applying exclusion criteria, 303 patients were included in the final analysis. Exclusion reasons are shown in the diagram.

This single-center, retrospective cross-sectional study consecutively enrolled hospitalized patients with Type 2 Diabetes Mellitus (T2DM) who underwent clinically indicated coronary computed tomography angiography (CCTA) at West China Hospital from January 2021 to October 2024. Written informed consent was obtained from all participants. Of the 485 patients initially identified, 303 satisfied the inclusion criteria and were incorporated into the final analysis (**Figure 1**).

The inclusion criteria were as follows: (1) diagnosis of T2DM according to the 2019 International Diabetes Federation guidelines; (2) completion of CCTA with diagnostic-quality imaging; and (3) availability of comprehensive laboratory data within one week prior to the CCTA procedure.

Exclusion criteria included: (1) current or previous use of statin therapy; (2) presence of severe hepatic or renal dysfunction, or uncontrolled thyroid disorders; (3) history of coronary revascularization; and (4) CCTA images of non-diagnostic quality.

### 2.2 Clinical data collection

Demographic characteristics, medical history, and laboratory data were collected from electronic health records. Body mass index (BMI) was computed by dividing body weight in kilograms by the square of height in meters. Smoking status was defined as a lifetime consumption of at least 100 cigarettes, encompassing both current smokers (those who smoked within the past six months) and former smokers (those who had ceased smoking for six months or longer). Hypertension was defined as blood pressure ≥140/90 mmHg or the use of antihypertensive medications. Fasting venous blood samples were obtained following an overnight fast of at least 8 hours. All laboratory parameters were measured by standard enzymatic methods using an automatic biochemical analyzer. The parameters included fasting plasma glucose, glycated hemoglobin (HbA1c), total cholesterol (TC), triglycerides (TG), high-density lipoprotein cholesterol (HDL-C), and low-density lipoprotein cholesterol (LDL-C).

The Atherogenic Index of Plasma (AIP) was calculated as the base-10 logarithm of the ratio of triglycerides to HDL-C [log10(TG/HDL-C)].

### 2.3 CCTA image acquisition and analysis

CCTA was performed utilizing either a 512-slice GE Revolution scanner or a 640-slice United Imaging uCT960+ scanner, following standardized imaging protocols. Retrospective electrocardiographic (ECG) gating was employed throughout image acquisition. Acquisition parameters included a tube voltage ranging from 100 to 120 kV, automatically adjusted according to patient BMI; tube current between 150 and 400 mAs; slice thickness of 0.5 to 0.6 mm; and a gantry rotation time of 0.25 seconds. The scanning field extended from 1 to 2 cm below the tracheal bifurcation to the diaphragmatic surface of the heart. Intravenous administration of contrast agent (Ultravist 370, Bayer) was performed at a rate of 5.0 mL/s, followed by a saline flush at 4.0 mL/s (20 mL), preceded by a test bolus of saline at 6.0 mL/s (20 mL). The total contrast volume ranged from 50 to 90 mL, corresponding to 1 to 1.5 mL/kg of body weight. Automatic triggering was used with a threshold of 100 HU in the ascending aorta. Only patients exhibiting sinus rhythm and stable heart rates were included. For those with elevated or irregular resting heart rates, β-receptor blockers were administered one hour prior to scanning. In the absence of hypotension and with heart rate <100 bpm, sublingual nitroglycerin was given 3–5 minutes before scanning.

Two experienced radiologists (with >5 years of work experience), blinded to all clinical and laboratory information, independently analyzed all CCTA images. Inter-reader agreement was excellent (weighted Cohen’s κ> 0.80). Disagreements were resolved by consensus. Coronary stenosis severity was quantified in all major epicardial vessels with a diameter ≥2.0 mm using semi-automated analysis software and graded according to established criteria (17): 0% (none), 1%–24% (minimal), 25%–49% (mild), 50%–69% (moderate), 70%–99% (severe), and 100% (occlusion). Stenosis severity was assessed per patient based on the most severe stenosis in any major epicardial coronary artery.

The primary outcome was obstructive coronary artery disease (CAD), defined as luminal stenosis of 50% or greater in any major epicardial coronary artery. This threshold aligns with the European Society of Cardiology (ESC) guidelines for clinically significant CAD. Additionally, stenosis of 70% or greater, categorized as severe is recognized as hemodynamically significant and frequently informs decisions regarding revascularization.

### 2.4 Statistical methods

The normality of data distributions was evaluated using the Shapiro-Wilk test. Normally distributed continuous variables are presented as mean ± standard deviation (SD), and non-normally distributed data as median [interquartile range, IQR]. Categorical variables are reported as frequencies (percentages). Group comparisons utilized ANOVA (with Tukey’s *post hoc* test) or Kruskal-Wallis test (with Dunn’s *post hoc* test) for continuous variables, and chi-square or Fisher’s exact test for categorical variables. Correlations between variables were assessed using Pearson’s or Spearman’s correlation coefficients, contingent upon data distribution. Missing data were minimal and assumed to be missing at random. Complete case analysis was conducted for all analyses.

Logistic regression analysis was employed to identify factors associated with obstructive CAD (stenosis ≥50%). The following variables were included in the multivariate model: age, sex, BMI, smoking status, hypertension, diabetes duration, HbA1c, total cholesterol, serum creatinine, and AIP (both as continuous variable and as quartiles). Variables with P < 0.10 in univariate analysis were included in a backward stepwise multivariate model (removal P > 0.05). Multicollinearity was assessed using variance inflation factors (VIF < 5). Model calibration was evaluated with the Hosmer-Lemeshow test.

The discriminative ability of lipid indices was evaluated using receiver operating characteristic (ROC) curve analysis, with optimal cut-offs determined by Youden’s index. Differences in area under the curve (AUC) were compared with DeLong’s test.

All analyses were performed using SPSS 27.0, R 4.3.2, and MedCalc 23.2.1, with a two-tailed P < 0.05 considered statistically significant.

## 3 Results

### 3.1 Baseline characteristics

A total of 303 patients with T2DM were included in the final analysis, with a mean age of 67.0 ± 10.3 years and a male predominance (67.7%). The median AIP of the cohort was 0.10 (IQR: -0.07 to 0.29). Using the quartile cut-points derived from the overall AIP distribution, patients were categorized into four groups: Q1 (AIP ≤ -0.072, n=76), Q2 (AIP -0.072 to 0.097, n=75), Q3 (AIP 0.097 to 0.301, n=77), and Q4 (AIP > 0.301, n=75).

The baseline demographic, clinical, and laboratory characteristics of the patients across the four AIP quartiles are presented in **Table 1**. Overall, there were no statistically significant differences among the quartile groups regarding age, sex distribution, prevalence of hypertension, systolic or diastolic blood pressure, duration of diabetes, smoking history, or alcohol consumption (all P > 0.05).

**Table 1:** Comparison of baseline characteristics across AIP quartiles.

Variable	Total population (n=303)	Q1 (n=76)	Q2 (n=75)	Q3 (n=77)	Q4 (n=75)	χ²/F/H	*P*-value
AIP*[M (IQR)]	0.10 (-0.07,0.29)	-0.20 (-0.33,-0.12)	0.03 (-0.02,0.06)	0.17 (0.13,0.23)	0.45 (0.35,0.58)^**abc**^	**28.312**	**<0.001**
Male,n (%)	205 (67.7%)	51 (67.1%)	46 (61.3%)	55 (71.4%)	53 (70.7%)	2.192	0.533
Female,n (%)	98 (32.3%)	25 (32.9%)	29 (38.7%)	22 (28.6%)	22 (39.3%)		
Age (years)	67.02 ± 10.30	68.35 ± 9.71	67.51 ± 10.65	65.12 ± 9.45	65.02 ± 10.30	1.388	0.246
BMI (kg/m²)	24.46 ± 3.58	23.67 ± 2.64	24.41 ± 2.70a	23.38 ± 3.21	25.45 ± 3.12^**ac**^	**6.409**	**<0.001**
SBP (mmHg)	132.29 ± 17.27	129.78 ± 19.54	132.55 ± 16.63	133.57 ± 16.28	132.2 ± 16.51	0.761	0.516
DBP (mmHg)	80.33 ± 12.99	79.55 ± 11.30	79.75 ± 16.11	82.35 ± 11.09	79.64 ± 12.94	0.833	0.476
Diabetes duration (years)	9.60 ± 7.56	9.71 ± 7.55	10.29 ± 8.10	10.31 ± 7.93	8.07 ± 6.47	1.431	0.234
Hypertension, n (%)	177 (58.4)	42 (23.7)	40 (22.6)	50 (28.2)	45 (25.4)	2.533	0.469
Smoking history, n (%)	108 (35.6)	22 (20.4)	21 (19.4)	34 (31.5)	31 (28.7)	6.887	0.076
Alcohol consumption, n (%)	95 (31.4)	26 (27.4)	17 (17.9)	28 (29.5)	24 (25.2)	3.830	0.280
HbA1c (%)	7.96 ± 1.73	7.30 ± 1.2	8.07 ± 2.0	8.27 ± 1.9	8.60 ± 1.9^**a**^	**2.859**	**0.038**
FBG mmol/L)	8.03 ± 3.66	6.99 ± 2.81	7.52 ± 3.01	8.67 ± 3.81^a^	8.91 ± 4.50^**a**^	**4.872**	**0.003**
Cr (μmol/L)	82.93 ± 58.66	84.20 ± 40.21	73.68 ± 15.38	75.88 ± 17.86	98.24 ± 46.10	**2.721**	**0.045**
CRP*[M (IQR)]	7.22 (3.02,22.10)	7.86 (2.10,30.50)	7.29 (3.48,9.16)	7.05 (4.49,16.10)	8.19 (2.26,32.30)	0.506	0.918
TG (mmol/L)	1.69 ± 1.19	0.89 ± 0.24	1.26 ± 0.27^a^	1.63 ± 0.40^ab^	2.98 ± 1.70^**abc**^	**78.161**	**<0.001**
TC (mmol/L)	4.28 ± 1.09	4.29 ± 1.05	4.00 ± 0.98	4.18 ± 0.95	4.63 ± 1.26^**b**^	**4.615**	**0.004**
HDL (mmol/L)	1.17 ± 0.36	1.54 ± 0.36	1.20 ± 0.23^**a**^	1.05 ± 0.2^**ab**^	0.94 ± 0.2^**abc**^	**87.477**	**<0.001**
LDL (mmol/L)	2.56 ± 0.89	2.51 ± 0.9	2.29 ± 0.8	2.54 ± 0.9	2.90 ± 0.9	1.737	0.159
nonHDL-C (mmol/L)	3.08 ± 1.06	2.76 ± 0.9	2.77 ± 0.8	3.03 ± 0.9	3.72 ± 0.9^**abc**^	**16.614**	**<0.001**

1.*indicates continuous variables identified as non-normally distributed by the Shapiro-Wilk test, presented as median (interquartile range, IQR). 2.Superscript letters denote significant between-group differences (Dunn's test for non-normal data or Tukey's HSD test for normal data, P < 0.05): a vs. Q1, P < 0.05; b vs. Q2, P < 0.05; c vs. Q3, P < 0.05. 3.CRP units: mg/L. 4.Bold values indicate statistical significance (P < 0.05).

In contrast, significant intergroup differences were observed in key metabolic parameters (all global P < 0.05). Specifically, one-way ANOVA revealed significant differences in body mass index (BMI, F = 6.409, P < 0.001), glycated hemoglobin (HbA1c, F = 2.859, P = 0.038), and fasting blood glucose (FBG, F = 4.872, P = 0.003). Similarly, the Kruskal-Wallis test indicated significant differences in triglycerides (TG, P < 0.001), total cholesterol (TC, P = 0.004), high-density lipoprotein cholesterol (HDL-C, P < 0.001), and non-HDL cholesterol (non-HDL-C, P < 0.001).

*Post-hoc* analyses demonstrated that the highest AIP quartile (Q4) consistently exhibited the most unfavorable metabolic profile. Compared to the lowest quartile (Q1), patients in Q4 had significantly higher BMI, poorer glycemic control (elevated HbA1c and FBG), and a more atherogenic lipid pattern characterized by markedly higher TG and lower HDL-C levels (all pairwise P < 0.05 after adjustment for multiple comparisons). A clear graded association was observed, with most adverse parameters showing a progressive increase (or decrease for HDL-C) from Q1 to Q4 (**Table 1**).

### 3.2 Association between AIP and coronary artery stenosis severity

#### 3.2.1 Comparison of stenosis severity distribution across AIP quartiles

Stratification of the study population into AIP quartiles (Q1-Q4) revealed a significant dose-dependent association with the severity of coronary artery stenosis (**Table 2**). The distribution of stenosis categories across AIP quartiles demonstrated a clear and progressive pattern.

**Table 2:** Comparison of coronary artery stenosis severity distribution across AIP quartiles [n (%)].

AIP quartile	No stenosis	Non-obstructive stenosis	Obstructive stenosis	Severe obstructive stenosis	Total
Q1	16 (21.1%)	33 (43.3%)	10 (13.2%)	17 (22.4%)	76 (100%)
Q2	13 (17.3%)	25 (33.3%)	22 (29.3%)	15 (20.0%)	75 (100%)
Q3	12 (15.6%)	20 (26.0%)	23 (29.9%)	22 (28.6%)	77 (100%)
Q4	19 (25.3%)	15 (20.0%)	15 (20.0%)	26 (34.7%)	75 (100%)

χ² = 19.74, P = 0.020 (Pearson’s chi-square test).

Patients in the lowest AIP quartile (Q1) had the highest proportion of non-stenotic lesions (21.1%) and predominantly exhibited non-obstructive stenosis (43.4%). With each increasing AIP quartile, there was a marked shift toward more severe disease. The prevalence of obstructive stenosis (≥50%) showed a progressive rise, while the proportion of non-obstructive stenosis declined correspondingly. Most strikingly, the highest AIP quartile (Q4) had the lowest rate of non-obstructive stenosis (20.0%) but the highest incidence of severely obstructive stenosis (≥70%; 34.7%).

These distribution patterns were confirmed to be statistically significant by chi-square testing (χ²=19.74, P = 0.020), indicating that higher AIP levels are strongly associated with more severe coronary artery stenosis.

#### 3.2.2 Correlation between continuous AIP and stenosis severity

**Figure 2:**
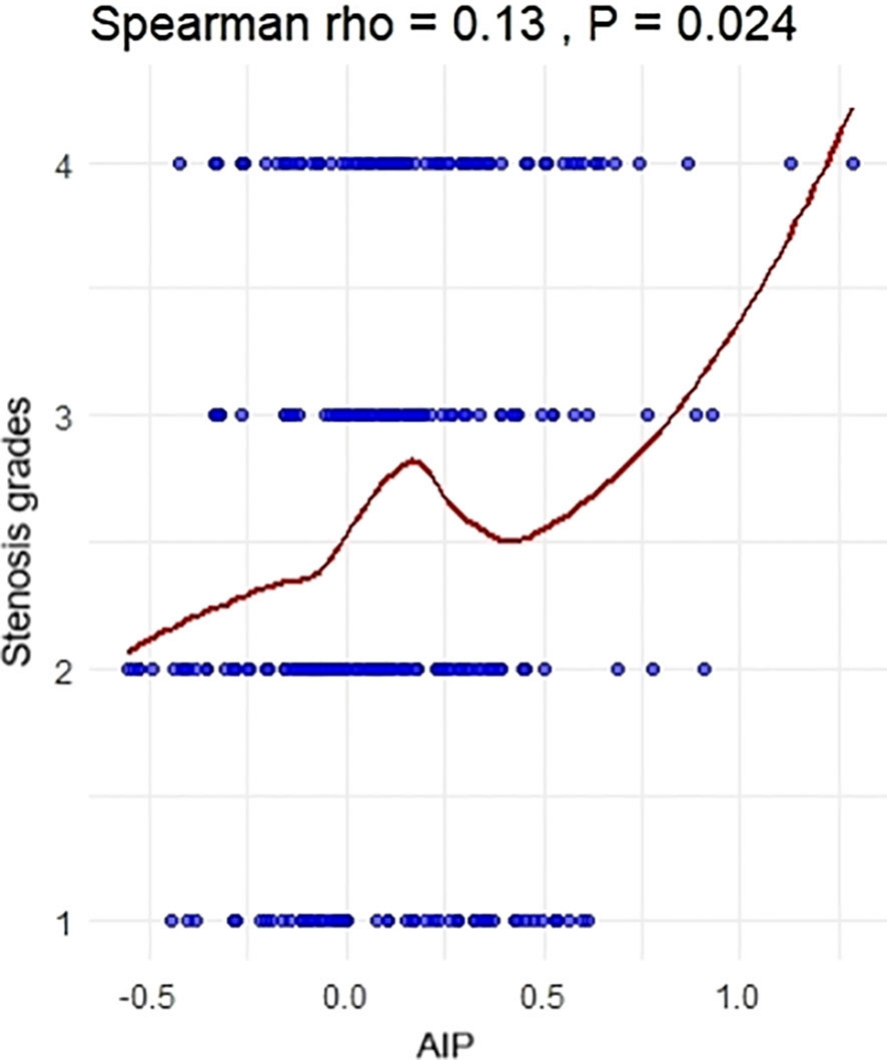
Scatter plot depicting the correlation between the atherogenic index of plasma (AIP) and the severity of coronary artery stenosis.

Spearman correlation analysis revealed a statistically significant, yet weak, positive correlation between AIP levels and the ordinal severity of coronary artery stenosis (ρ = 0.13, P = 0.024). This relationship is visually summarized in **Figure 2**, which presents a scatter plot of individual AIP values across the four stenosis severity categories. To enhance clarity, data points are horizontally jittered within each category. A fitted linear regression line with a 95% confidence band illustrates the overall positive trend of increasing AIP with higher stenosis grades. The distribution and trend observed in this continuous analysis are consistent with the significant dose-dependent association identified in the categorical quartile analysis (Section 3.2.1).

#### 3.2.3 Comparison of AIP levels across coronary stenosis severity groups

AIP levels differed significantly across the four coronary stenosis severity groups (Kruskal-Wallis H = 13.274, df = 3, P = 0.004). *Post-hoc* Dunn’s tests with Bonferroni correction were performed, using the Non-obstructive stenosis group as the reference.

As detailed in **Table 3**, median AIP exhibited a progressive increase with stenosis severity. The Severe obstructive stenosis group had the highest median AIP (0.14), which was significantly greater than the reference group (adjusted P < 0.001). The Obstructive stenosis group also had a significantly higher median AIP (0.11) than the reference (adjusted P = 0.012). The difference between the No stenosis group (median AIP: 0.10) and the reference group did not remain statistically significant after correction for multiple comparisons (adjusted P = 0.045, ns).

**Table 3:** Comparison of AIP levels across coronary artery stenosis severity categories.

Stenosis severity	n	Median (IQR)	*P-value*	H (df)	Pairwise comparison vs reference (Bonferroni-adjusted P-value)
No stenosis	60	0.10 (-0.09,0.35)	0.004	H(3)=13.274	**0.045**
Non-obstructive stenosis (Reference)	93	0.05 (-0.15,0.23)			
Obstructive stenosis	70	0.11 (-0.00,0.27)			**0.012**
Severe obstructive stenosis	80	0.14 (-0.12,0.35)			**<0.001**

Bold values indicate statistical significance (P < 0.05).

#### 3.2.4 Association of AIP with obstructive coronary artery disease

Binary logistic regression models were employed to evaluate predictors of obstructive CAD (stenosis ≥50%). Univariate analysis (**Table 4**) revealed that elevated AIP levels (OR = 3.74, 95% CI: 1.66–8.43, P = 0.001) and longer diabetes duration (OR = 1.039, 95% CI: 1.007–1.071, P = 0.017) were associated with increased odds. Notably, smoking history was associated with a reduced odds (OR = 0.610, 95% CI: 0.380–0.981, P = 0.041), a finding that requires careful interpretation.

**Table 4:** Analysis of risk factors associated with coronary artery stenosis (≥50%).

Variable	Stenosis<50% (n=153)	Stenosis ≥50% (n=151)	Exp (B)	95%CI	*P*-value
AIP	0.13 ± 0.26	0.15 ± 0.15	3.740	1.660-8.428	**0.001**
Age (years)	62.13 ± 10.58	66.80 ± 9.75	1.020	0.997-1.043	0.085
Male,n (%)	97 (63.4%)	108 (71.5%)	0.674	0.415-1.094	0.110
Female,n (%)	56 (36.6%)	43 (28.5%)			
BMI (kg/m²)	24.73 ± 3.81	23.24 ± 3.22	0.971	0.910-1.037	0.380
SBP (mmHg)	128.08 ± 19.31	134.92 ± 20.56	1.009	0.996-1.023	0.168
DBP (mmHg)	79.21 ± 13.71	80.36 ± 12.81	0.989	0.972-1.007	0.230
Diabetes duration (years)	7.86 ± 6.56	12.52 ± 8.22	1.039	1.007-1.071	**0.017**
Hypertension,n (%)	84 (54.9)	93 (62)	0.746	0.472-1.180	0.211
Alcohol use, n (%)	41 (26.8)	54 (36)	0.651	0.399-1.061	0.085
Smoking, n (%)	46 (30.1)	62 (41.3)	0.610	0.380-0.981	**0.041**
HbA1c (%)	8.28 ± 1.63	8.50 ± 1.92	0.921	0.789-1.074	0.295
FBG mmol/L)	9.39 ± 5.04	8.47 ± 3.70	0.977	0.917-1.040	0.458
Creatinine (μmol/L)	80.96 ± 41.45	82.24 ± 19.94	1.003	0.997-1.008	0.310
CRP (mg/L)	18.25 ± 29.75	17.32 ± 34.53	1.003	0.990-1.016	0.668

Binary logistic regression analysis was performed with continuous variables entered directly into the model. For categorical variables, the last category was designated as the reference group. Bold values indicate statistical significance (P < 0.05).

After adjusting for demographic variables (age and sex) in Model 1, AIP remained significantly associated (adjusted OR = 3.40, 95% CI: 1.85–10.34, P = 0.007), though the wide confidence interval indicates imprecision in the point estimate. This association persisted in models with further adjustments (Models 2, 3, **Table 5**).

**Table 5:** Multivariate logistic regression analysis of risk factors for coronary artery stenosis (≥50%) using stepwise selection method.

Variable	Model 1		Model 2		Model 3	
	OR (95%CI)	*P*-value	OR (95%CI)	*P*-value	OR (95%CI)	*P*值
Gender (Male vs Female)	0.646 (0.348-1.203)	0.169				
Age	1.025 (0.998-1.072)	0.066				
SBP	1.011 (0.994-1.028)	0.225	1.008 (0.993-1.002)	0.289		
Hypertension	0.832 (0.492-1.404)	0.490	0.817 (0.495-1.348)	0.428		
Smoking	0.688 (0.378-1.250)	0.220	0.603 (0.365-0.996)	0.480	0.607 (0.368-1.000)	0.050
Diabetes duration	1.039 (1.005-1.075)	**0.025**	1.045 (1.012-1.078)	**0.008**	1.047 (1.014-1.081)	**0.005**
AIP	4.404 (1.853-10.342)	**0.007**	3.837 (1.656-8.890)	**0.002**	3.931 (1.700-9.623)	**0.001**

Model 1: Adjusted for sex, age, systolic blood pressure (SBP), hypertension history, smoking history, diabetes duration, and AIP. Model 2: Adjusted for SBP, hypertension, smoking history, diabetes duration, and AIP. Model 3: Adjusted for smoking history, diabetes duration, and AIP. All categorical variables used the second category as reference (e.g., female as reference for sex). Bold values indicate statistical significance (P < 0.05).

**Figure 3:**
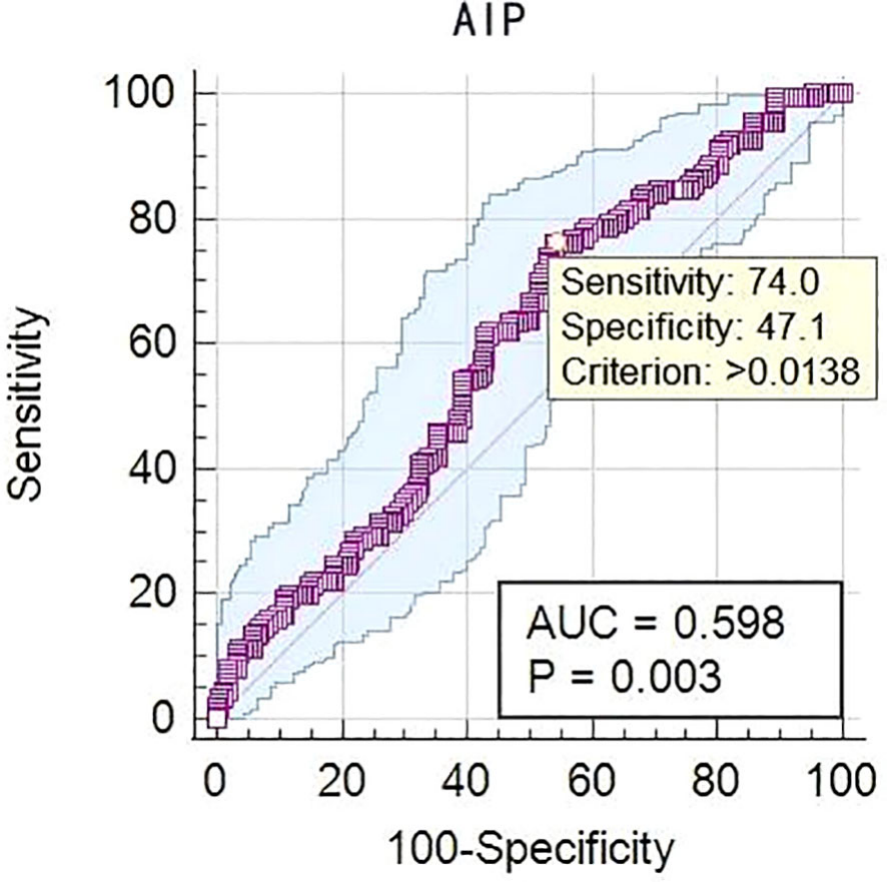
Receiver Operating Characteristic (ROC) curve of AIP for discriminating Coronary Artery Stenosis ≥50%.

**Figure 4:**
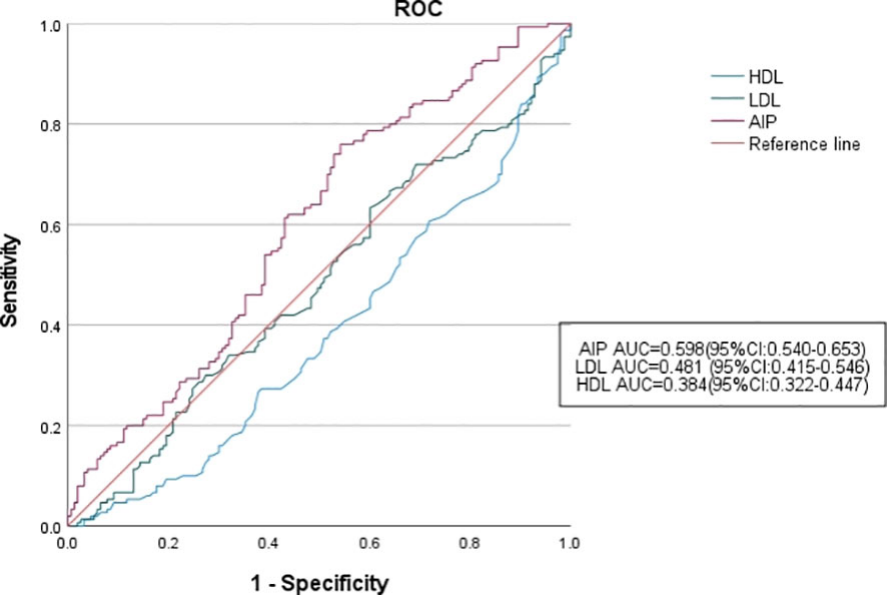
Comparative ROC Curve Analysis of AIP, LDL-C, and HDL-C for discriminating Coronary Artery Stenosis ≥50%.

ROC curve analysis indicated that AIP had a significant but modest discriminatory ability for obstructive CAD, with an AUC of 0.598 (95% CI: 0.540–0.653, P = 0.002) (**Figure 3**). The optimal cutoff was 0.0138 (sensitivity 74.0%, specificity 47.1%). Comparative analysis showed that the AUC of AIP was statistically significantly higher than that of LDL-C (AUC = 0.481, P = 0.032) and HDL-C (AUC = 0.384, P < 0.001) (**Figure 4**).

The atherogenic index of plasma (AIP) demonstrated statistically significant discriminative ability for coronary artery stenosis ≥50%, with an area under the curve (AUC) of 0.598 (95% confidence interval [CI]: 0.540-0.653; P = 0.003). At the optimal cutoff value of 0.014 determined by Youden’s index, the model achieved a sensitivity of 74.0% and specificity of 47.1%, yielding a Youden index of 0.218.

The AIP curve (solid red line) was positioned closest to the top-left corner, indicating superior diagnostic performance, followed sequentially by LDL-C (solid green line) and HDL-C (solid blue line).

The area under the curve (AUC, 95% confidence interval) values were:AIP: 0.598 (0.540–0.653), LDL-C: 0.481 (0.415–0.546), HDL-C: 0.384 (0.322–0.447).DeLong’s test confirmed that AIP had significantly higher discriminative ability than:LDL-C (Z = 2.14, P = 0.032), HDL-C (Z = 3.87, P < 0.001).

## 4 Discussion

This study represents an evaluation of the relationship between the Atherogenic Index of Plasma (AIP) and coronary artery stenosis severity in patients with type 2 diabetes mellitus (T2DM), providing a biomarker option for early warning of diabetic cardiovascular complications. The results not only confirmed a positive correlation between AIP and coronary stenosis severity (r = 0.13, P < 0.05), but also importantly revealed a dose-response relationship between AIP levels and the severity of coronary lesions, underscoring its clinical significance.

From a pathophysiological standpoint, patients categorized within the highest AIP quartile (Q4) exhibited more severe metabolic disturbances, including higher BMI, HbA1c, fasting plasma glucose, and triglyceride levels, as well as lower HDL-C levels (all P < 0.05). This finding supports the theory proposed by Dobiášová (18) that AIP reflects abnormal lipoprotein particle quality. Recent studies have demonstrated that elevated AIP indicates an increase in small, dense LDL particles, which are more susceptible to oxidation and vascular endothelial penetration, thereby initiating the atherosclerotic process (19). Importantly, in this study, AIP maintained its independent association with coronary artery stenosis of 50% or greater (odds ratio = 3.931; 95% confidence interval: 1.700–9.623) even after adjustment for conventional cardiovascular risk factors, including age, sex, hypertension, and smoking status. This outcome is consistent with findings reported by Zheng Q et al. (20) and Fernández-Macías et al. (21) in populations with metabolic syndrome, suggesting that AIP may contribute to atherosclerosis through non-traditional pathways, such as promoting vascular inflammation and endothelial dysfunction (22).

These pathophysiological observations further support the potential clinical utility of AIP for cardiovascular risk stratification in T2DM patients. In terms of clinical application, receiver operating characteristic (ROC) analysis demonstrated that AIP achieved an area under the curve (AUC) of 0.598 (95% CI: 0.540–0.653) for identifying obstructive coronary artery disease. Although this represents modest discriminatory ability, it was statistically superior to that of traditional lipid indicators including LDL-C (AUC = 0.481) and HDL-C (AUC = 0.384) (both P < 0.05). This superiority of AIP over conventional lipid parameters may be explained by its ability to simultaneously reflect the balance between atherogenic and anti-atherogenic lipoproteins (23, 24). The association between AIP and coronary artery disease observed in our study is supported by a recent umbrella review of meta-analyses (25), which reported that higher AIP was significantly associated with CAD (OR = 2.74, 95% CI: 2.05–3.66, P < 0.01). However, the discriminative ability of AIP in our cohort (AUC = 0.598) was lower than that reported in some previous studies. This discrepancy may be attributable to the higher baseline cardiovascular risk profile and the exclusion of statin-treated individuals within our diabetic cohort, as well as variations in disease prevalence across different study populations. Despite these considerations, the overall limited discriminative ability suggests that AIP alone is insufficient as a primary screening tool but may serve as a valuable adjunctive risk marker. Furthermore, in comparison to studies conducted by Wu X et al. and Han H et al. (26, 27) involving the general population, the optimal AIP cutoff identified in our diabetic cohort (0.0138) was notably lower. This difference may reflect the impact of the diabetic lipid triad—low HDL-C, hypertriglyceridemia, and increased small, dense LDL—on the AIP threshold (28). Collectively, these findings imply that lower AIP thresholds may be considered for risk assessment in diabetic patients.

An important finding of this study is the clear graded association between coronary artery stenosis severity and AIP levels. Specifically, the median AIP value (0.14) observed in the group with severe obstructive stenosis (≥70%) was significantly elevated compared to other groups (P < 0.001), with AIP levels exhibiting a progressive increase corresponding to the worsening degree of stenosis. This result is broadly consistent with the study by Kosmas et al. (29, 30), who reported a positive correlation between AIP and coronary plaque burden. Notably, in this study, even the non-stenosis group had a median AIP level (0.10) significantly higher than reference values for healthy individuals (31). This finding may reflect the residual cardiovascular risk commonly observed in patients with T2DM (32), indicating a persistent risk of atherosclerosis progression even when LDL-C levels are at target.

In the context of residual cardiovascular risk despite LDL-C control, the findings of this study provide novel insights into cardiovascular risk management among diabetic populations. Traditional lipid-lowering strategies primarily focus on achieving LDL-C targets; however, this study suggests that simultaneously monitoring the triglyceride to HDL-C ratio, as represented by AIP, may more effectively identify individuals at elevated risk. Supporting this notion, subgroup analysis of the ACCORD lipid study (33) demonstrated that adding fenofibrate to statin therapy provided greater cardiovascular benefits for patients with high triglycerides and low HDL-C levels, which aligns with our findings. Furthermore, recent research indicates that risk assessment models based on AIP trajectories can more accurately predict cardiovascular event risk (34). This suggests that dynamic monitoring of AIP is clinically more valuable than a single measurement, as it reflects individual responses to lipid-lowering therapy and changes in metabolic status over time.

The findings of this study also raise several questions worthy of further investigation. First, the correlation between AIP and coronary stenosis, while statistically significant, was modest (r = 0.13, P = 0.024). This likely reflects the multifactorial nature of atherosclerosis, where stenosis severity is influenced not only by lipid abnormalities but also by plaque composition, calcification, vessel remodeling, and inflammatory processes. Importantly, despite the weak bivariate correlation, multivariable logistic regression demonstrated a strong independent association (OR = 3.93), suggesting that AIP contributes to stenosis risk in a manner not fully captured by linear correlation. Second, the specificity of the AIP risk assessment model in this study was relatively low (47.1%), highlighting the need to develop a composite scoring system that integrates AIP with other risk factors to improve diagnostic accuracy. Finally, the potential role of AIP as a novel therapeutic target in diabetes remains to be established and requires validation through large-scale interventional studies.

## 5 Limitations

The limitations of this study should be considered when interpreting the results:

1. The cross-sectional study design precludes the establishment of causal relationships between AIP and coronary artery stenosis; thus, all observed associations should be regarded as correlational rather than indicative of causality.
2. The relatively modest sample size restricts the capacity for detailed subgroup analyses, such as stratification by varying durations of diabetes mellitus.
3. The exclusion of patients receiving statin therapy limits the external validity of the results, particularly given the widespread use of statins in real-world diabetic populations. Moreover, the potential influence of non-statin lipid-lowering agents on AIP levels was not examined.
4. Coronary artery stenosis was assessed using contrast-enhanced imaging techniques rather than more precise methods such as intravascular ultrasound (IVUS) or optical coherence tomography (OCT) which may provide superior characterization of plaque morphology.
5. Detailed information on glucose-lowering medications (e.g., metformin, insulin) and antihypertensive agents was not systematically collected in this retrospective cohort, which may confound the observed associations between AIP and coronary stenosis.

## 6 Future directions

Based on the current findings, several avenues for future research merit consideration:

1. Future prospective cohort studies are needed to validate the association between AIP and hard cardiovascular endpoints (e.g., myocardial infarction, cardiovascular death) in T2DM patients, as our cross-sectional design precludes causal inference.
2. Multicenter investigations incorporating statin-treated individuals and ethnically diverse populations should be undertaken to evaluate the generalizability of these findings beyond single-center, statin-naïve Chinese cohorts.
3. Future research should evaluate whether AIP adds incremental value to existing risk scores (e.g., Framingham Risk Score, UKPDS Risk Engine) or coronary artery calcium scoring (CACS) for improved risk stratification.
4. Interventional trials are warranted to determine if AIP-guided therapeutic strategies, including intensified lipid-lowering interventions or lifestyle modifications, can improve clinical outcomes in diabetic patients.
5. Longitudinal research examining serial measurements of AIP over time may yield greater prognostic insight than single time-point assessments, as dynamic changes in AIP could reflect individual responses to treatment.
6. Incorporation of multimodal imaging techniques, such as IVUS and OCT, is recommended to more accurately elucidate the relationship between AIP and plaque characteristics, moving beyond reliance on luminal stenosis alone.

## 7 Conclusion

In summary, AIP is independently associated with the presence of ≥50% coronary stenosis in patients with T2DM and demonstrated superior discriminative capacity compared to conventional lipid parameters within this cohort.

AIP encapsulates the hallmark features of diabetic dyslipidemia, characterized by elevated triglycerides, reduced HDL-C, and increased small dense LDL.

The diabetes-specific AIP cutoff value identified (0.0138), along with elevated AIP levels observed even in patients without stenosis detected by coronary computed tomography angiography (CCTA), underscores its potential utility in identifying residual cardiovascular risk.

Further prospective studies are warranted to validate whether AIP-guided strategies might help guide risk management and improve clinical outcomes in diabetic populations.

## Data Availability

The raw data supporting the conclusions of this article will be made available by the authors, without undue reservation.
